# Superiority of Classification Tree versus Cluster, Fuzzy and Discriminant Models in a Heartbeat Classification System

**DOI:** 10.1371/journal.pone.0140123

**Published:** 2015-10-13

**Authors:** Vessela Krasteva, Irena Jekova, Remo Leber, Ramun Schmid, Roger Abächerli

**Affiliations:** 1 Institute of Biophysics and Biomedical Engineering, Bulgarian Academy of Sciences, Sofia, Bulgaria; 2 Biomed Research and Signal Processing, Schiller AG, Baar, Switzerland; 3 Bern University of Applied Sciences, Medical Technology Center, Bern, Switzerland; 4 University Hospital Basel, Cardiovascular Research Institute Basel, Bazel, Switzerland; Universidad de Valladolid, SPAIN

## Abstract

This study presents a 2-stage heartbeat classifier of supraventricular (SVB) and ventricular (VB) beats. Stage 1 makes computationally-efficient classification of SVB-beats, using simple correlation threshold criterion for finding close match with a predominant normal (reference) beat template. The non-matched beats are next subjected to measurement of 20 basic features, tracking the beat and reference template morphology and RR-variability for subsequent refined classification in SVB or VB-class by Stage 2. Four linear classifiers are compared: cluster, fuzzy, linear discriminant analysis (LDA) and classification tree (CT), all subjected to iterative training for selection of the optimal feature space among extended 210-sized set, embodying interactive second-order effects between 20 independent features. The optimization process minimizes at equal weight the false positives in SVB-class and false negatives in VB-class. The training with European ST-T, AHA, MIT-BIH Supraventricular Arrhythmia databases found the best performance settings of all classification models: Cluster (30 features), Fuzzy (72 features), LDA (142 coefficients), CT (221 decision nodes) with top-3 best scored features: normalized current RR-interval, higher/lower frequency content ratio, beat-to-template correlation. Unbiased test-validation with MIT-BIH Arrhythmia database rates the classifiers in descending order of their specificity for SVB-class: CT (99.9%), LDA (99.6%), Cluster (99.5%), Fuzzy (99.4%); sensitivity for ventricular ectopic beats as part from VB-class (commonly reported in published beat-classification studies): CT (96.7%), Fuzzy (94.4%), LDA (94.2%), Cluster (92.4%); positive predictivity: CT (99.2%), Cluster (93.6%), LDA (93.0%), Fuzzy (92.4%). CT has superior accuracy by 0.3–6.8% points, with the advantage for easy model complexity configuration by pruning the tree consisted of easy interpretable ‘if-then’ rules.

## Introduction

Early detection of cardiac arrhythmias is potentially life-saving as any disturbance in the rate, regularity, site of origin or conduction of electrical impulses through the myocardium might refer to a structural or functional heart disease with the risk of developing heart failure [1]. Automatic detection and classification of heartbeats is an important computerized diagnostic tool, assisting cardiologists in the task of careful expert inspection of long-term electrocardiogram (ECG) recordings, marking the presence of sustained or casual (e.g. transient, short-term or infrequent) arrhythmias. Considering the inter-patient and intra-patient variation of the ECG waveform, some beat classification systems aim to improve their performance by taking the advantage from a local expert assistance for initial annotation of a group of typical or pathological beats in one ECG recording rather than only relying on a global learning strategy [2–7].

The analysis of the P-QRS-T waveform complexity and regularity of the cardiac cycle duration (RR-interval) is used for extraction of a diverse set of features which are then subjected to optimization in different decision support systems aiming at the most reliable classification of normal or abnormal beats with supraventricular or ventricular origin. The feature extraction techniques can be grouped according to the mathematical models used to assess the P-QRS-T waveform complexity. The widely used approach applies P-QRS-T onset/offset delineation or extraction of QRS patterns within a fixed-length window around the fiducial point to measure morphological features in the time domain, including amplitudes, areas, specific interval durations or magnitudes and angles of the QRS vectors in the vectorcardiographic (VCG) planes [2–5,8–19]. Other ECG descriptors rely on QRS frequency components calculated either by discrete Fourier transform (DFT) [11,18,20] or by computationally efficient algorithms with filter banks [4,21]. The waveform of fixed-length QRS patterns is analyzed either in the time domain by cross-correlation with a reference beat template [4,22] or in the time-frequency domain by decomposition to large-scale basis functions and extraction of their coefficients by discrete wavelet transform (DWT) [10,15,18,19,23–25], wavelet packet decomposition (WPD) [11,26], Matching Pursuits [5,27], Hermite basis functions [10,28–30], principal-component analysis (PCA), referred also as Karhunen-Loève transform [2,6,23,31]. The dynamic ECG features estimated as a variation of the neighboring RR-intervals are considered in almost all published works.

Different mathematical approaches for decision support systems have been proposed for the automatic classification of heartbeats. Widely applied classification methods are based on linear programming using the Kth nearest-neighbours (KNN) using clustering technique [5,9–11,26], linear discriminant analysis (LDA) [3,13–15], fuzzy analysis [4,12,21] and decision tree classifiers [7,8,16,17,21,25,32]. Another frequently used classifier is the support vector machine (SVM)–least square SVM applying linear kernel function [22–24] or SVM relying on quadratic optimization by mapping of the feature space into a high dimensional space using various kernel transformations like hyperbolic tangent sigmoid transfer function [18] or Gaussian radial basis function [19,20,23,24]. Artificial neural networks (ANNs) are also employed for heartbeat classification [2,23,27–29,31], although ANNs involve time consuming complex computations in the application phase, masking the features which are useful or worthless and the net decision making [32]. The latter study underlines the decision tree among a set of 16 classifiers, such as ANNs, nearest-neighbours, kernel density, etc. as the most accurate, the most time efficient, very flexible and easily interpretable representation language. The good performance of the decision tree is also demonstrated in a recent study [30], which combines a set of individual neural classifiers working in parallel and a subsequent decision tree to integrate the results, thus reporting about 9.5% lower error than in the case of the runner-up method of integration using the weighted voting mechanism. Decision trees are also well employed in the final classification step of a complex features extraction scheme by DWT, PCA and independent component analysis [25]. Such complicated computation schemes, however, are losing the benefit from simplicity that gains the decision tree model, which is the core for building remote automated real-time ECG analysis modules, such as PDAs (Personal Digital Assistants) [32] or wireless Holter monitors [7,8].

The existence of redundant feature vectors affects the performance of the classifier if not appropriately handled. Reduction of the feature space dimension by excluding irrelevant features carrying conflicting, duplicating or little information to the classifier is applied by means of higher order statistics [26], genetic algorithm [11,20], perturbation method [19], fuzzy c-means clustering [31], or Hermite function decomposition [28].

The objective of this work is to develop and compare the best performance of four independent realizations of linear programming beat classifiers based on cluster analysis, LDA, fuzzy analysis and classification tree (CT), all subjected to iterative training for selection of the optimal feature space. The general concepts followed during the development, training and test process are:

- Computationally efficient and robust real-time beat classifier, without the need for local expert intervention, mandatory in fully automated monitoring devices for cardiac patients.
- Beat class labeling according to the ANSI/AAMI EC57 standard [33].
- Better generalization properties of the optimal feature set by multidatabase training approach.
- Unbiased performance evaluation on independent training [34–36] and test [37] ECG databases which are a common standard for inter- and intra-study comparisons.
- Input feature space formed by a set of basic features with a physiological meaning, tracking the morphology and RR-interval variation, and correlation to noise robust average beat templates.
- Iterative training for selection of the optimal feature space, aiming at each step to minimize the number of false positive and false negative errors.

## ECG Databases

The study involves all full-length recordings in four ECG databases owing internationally recognized heartbeat annotations of many common and life-threatening arrhythmias:

- АHА –AHA database [34]: 80 ECG recordings, 2 leads, 30min in duration;
- EDB–European ST-T database [35]: 90 ECG recordings, 2 leads, 2h;
- SVDB–MIT-BIH Supraventricular Arrhythmia Database [36]: 78 ECG recordings, 2 leads, 30min;
- MIT-BIH–MIT-BIH Arrhythmia Database [37]: 48 ECG recordings, 2 leads, 30min.

ECG signals are processed with a common sampling rate of 250Hz. EDB and AHA keep their original sampling rate (250Hz), while MIT-BIH (360Hz) and SVDB (125Hz) are linearly interpolated to 250Hz. Filtering in a bandwidth 0.05–75Hz is applied, although, signals could be already more band-limited within the databases. Two composite leads are next analyzed:

$$\text{Magnitude}:\;mag=\sqrt{lead1^{2}+lead2^{2}}\;;\;\text{Velocity}:\;vel=\sqrt{{\left(\Delta lead1\right)}^{2}+{\left(\Delta lead2\right)}^{2}}\;,$$

where Δlead1 and Δlead2 are the first order derivatives of lead1 and lead2, estimated as the difference between two neighboring lead samples on the time scale.

An automatic heartbeat detector is run and the correctly identified QRS complexes which can be paired with the database reference beat annotation labels within a window of 150ms are further included in the heartbeat classification study. The original database beat annotation labels are converted to one of the five beat types recommended by ANSI/AAMI EC57:1998 standard [33]:

- N-beat: Sinus node beat, including normal beat, left and right bundle branch block;
- S-beat: Supraventricular ectopic beat, including an atrial or nodal (junctional) premature or escape beat, or an aberrated atrial premature beat;
- V-beat: Ventricular ectopic beat, including a ventricular premature beat, R-on-T ventricular premature beat, or ventricular escape beat;
- F-beat: Fusion of ventricular and normal beat;
- Q-beat: Unclassified beat, including unknown beat, paced beat, a fusion of paced and normal beat.
- Two general heartbeat classes are defined in the beat classification task:
- SVB-class: the class of beats with supraventricular origin (N+S beats);
- VB-class: the class of beats with ventricular origin (V+F beats).

This generalization to a binary class model (SVB and VB) is a clinically relevant pre-step of the beat type (N,S,V,F) classification problem that distinguishes between beats of normal morphology (narrow supraventricular beats) and abnormal morphology (dangerous wide ventricular beats).

Independent datasets are used for training (AHA, EDB, SVDB) and test-validation (MIT-BIH) with a total duration of about 300h and 24h, respectively. Table 1 shows the number of annotated beats which have been analyzed in each database.

**Table 1:** Sample size, including the total number of N, S, V, F beats in ECG databases.

	Supraventricular beats (SVB-class)		Ventricular beats (VB-class)	
Beat Annotation	N beats	S beats	V beats	F beats
EDB–Training	784633	1100	4467	354
SVDB–Training	162340	12198	9943	23
AHA–Training	160585	0	16496	829
MIT-BIH–Test	90388	3025	7235	803

## Methods

**Fig 1:**
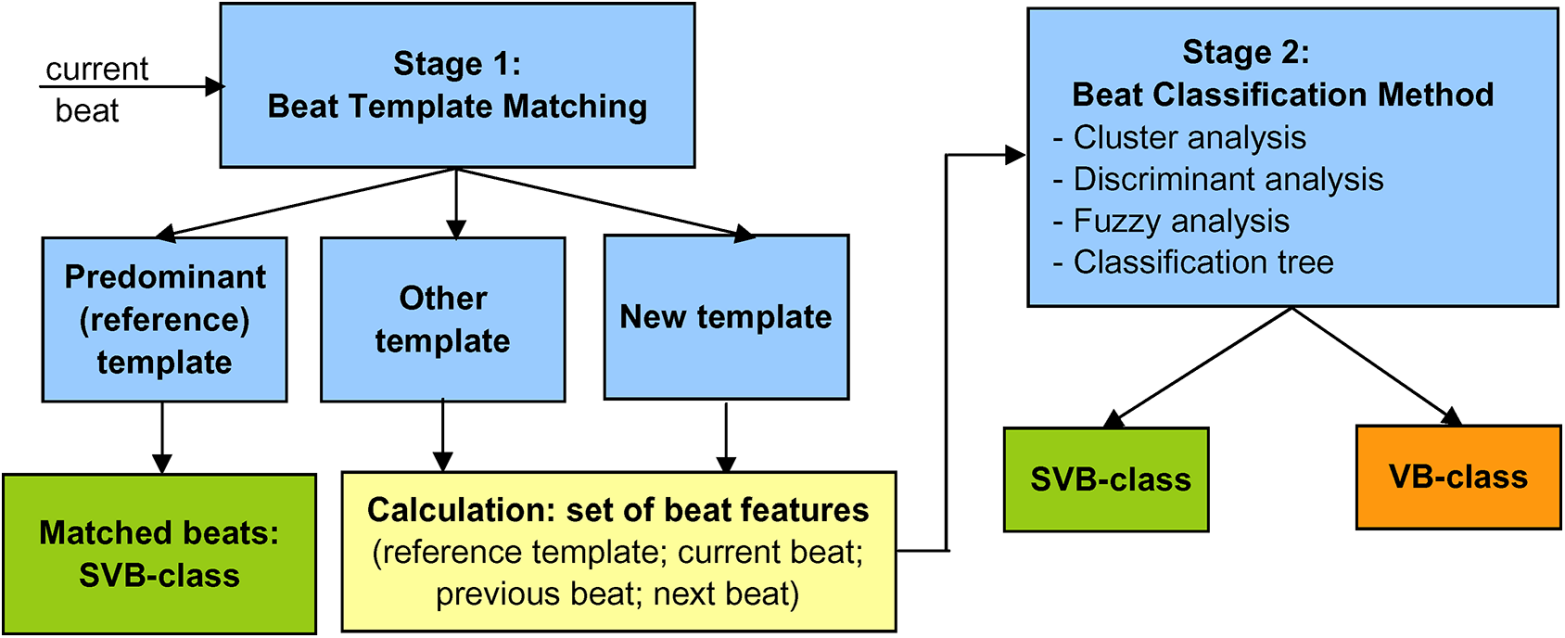
Concept diagram of the two-stage beat classifier.

The presented beat classifier is based on a two-stage decision system (Fig 1), applying initial fast assignment of beats to SVB-class, close matching the reference beat template of the patient’s predominant rhythm (Stage 1), and a subsequent classification of the non-matched beats to SVB or VB-class (Stage 2). Different classification methods which allow implementation of a real-time processing concept are studied as a Stage 2 beat classifiers, including Cluster, Fuzzy, Discriminant and Classification tree models. The next subsections briefly describe the background of each method.

### Stage 1: Beat template matching

Stage 1 is a kind of PQRST waveform preprocessor, which accumulates average beat templates, makes template matching for fast assignment of beats in SVB-class and calculates a set of features only for the non-classified beats.

During the learning period (initial 10s of the recording), the predominant normal (reference) beat template is created. For this purpose, all heartbeats within the learning period are assigned to subgroups of similar morphologies. The number of members and the QRS duration of each subgroup are used in a weighted combination for finding the largest subgroup with the shortest QRS duration. Averaging of all beats in this subgroup defines the predominant normal beat template, which is compared to each further beat.

The embedded template matching algorithm relies on correlation between the beat and the template waveforms. The template matching condition requires a correlation above an adaptive correlation threshold (ACT), which may vary between 80% and 98%. ACT is slowly adapted to the current noise conditions every 10s:

ACT(i)=0.75*ACT(i-1)+0.25*OCT(i-1),

where: *i* is the index of non-overlapping 10s segments; OCT is the optimal correlation threshold–the highest possible threshold value which fulfills the basic rule: at least 75% of beats in a 10s segment must match at least 25% of the other beats in the same segment. The intention is that OCT is set in the way that not every beat is in its own subgroup (too high threshold) and that not all the beats are always in the same subgroup (too low threshold). We expect that at least 75% of the beats can be assigned to not more than 4 different subgroups. The remaining 25% of the beats may be random, e.g. artefacts. OCT is calculated every 10s using all beats within the previous 10s segment and by scanning from 98% downwards in steps of 0.5% until the basic rule is fulfilled or the minimal value of 80% is reached. This procedure has been proven to be effective to follow changing noise conditions in many recordings from different patients.

During the test phase, each beat is compared to the reference template and if matching is found then the current beat is assumed to resemble the patient’s sustained rhythm, it is assigned to SVB-class and the reference template is dynamically updated. If the current beat does not match the reference template, then matching to other beat templates is verified and the matched one is updated. Otherwise a new template is created. The other beat templates are created for repetitive beat morphologies that do not match the predominant normal (reference) beat template. They help to distinguish a repetitive beat pattern from a random pattern (e.g. monomorphic ventricular beats vs. polymorphic ventricular beats or artefacts). A limited number of templates is supported (e.g. up to 8 templates) in order to save computation time in a real-time environment which is started and running for an undetermined amount of time. The correlation calculation requires many operations and the number of calculated correlation coefficients for every new beat increases proportionally with each additional beat template. Non-matched beats create a new template only if the limit of templates is not exceeded or may replace an existing template. Replacement of templates is just an option for a long-term acquisition, but it has not actually been done in the training and testing datasets. A possible strategy may be the replacement of the template with the smallest number of beat members that has not been hit for the longest time.

Some potential distinguishing properties of SVB vs. VB beats are well-known and were used as prior knowledge when creating the basic feature set with a related physiological meaning: P-wave existence (P wave is usually not present for VB-class), morphological similarity with the predominant reference template (typical for SVB-class), QRS complex properties (larger QRS duration/area, lower QRS frequency content are typical for VB-class), relative beat timing (VB beats occur earlier in time than the next expected beat). Stage 1 calculates a set of 20 basic features only for the beats not assigned to SVB-class, considering the time-domain behavior of the current beat, the neighboring beats (previous, next), and the noise robust reference template:

- 3 ternary discrete features, indicating if the current, previous and next beats are: matching the reference template (0), matching another beat template (1) or not matching any beat template (-1).
- 2 binary discrete features, indicating if the current beat and the reference template have a P-wave (no = 0 or yes = 1).
- 3 correlation coefficients, showing the waveform similarity of the current beat, the previous beat and the next beat against the reference template. The correlation (corr) is estimated from the composite velocity lead of a single beat (Bvel) and beat template (Tvel) within 180 ms window after the QRS onset:
  $$\text{corr}=100\frac{\sum_{\text{i}=\text{QRSon}}^{\text{QRSon}+180\;\text{ms}}{\text{Bvel}}_{\text{i}}\cdot {\text{Tvel}}_{\text{i}}}{\sqrt{\sum_{\text{i}=\text{QRSon}}^{\text{QRSon}+180\;\text{ms}}{\text{Bvel}}_{\text{i}}^{2}\cdot \sum_{\text{i}=\text{QRSon}}^{\text{QRSon}+180\;\text{ms}}{\text{Tvel}}_{\text{i}}^{2}}},(\%)$$
- 3 QRS durations (QRSdur) of the current beat, the reference template and the difference between both, where QRSdur is defined between the QRS boundaries:
  $$\text{QRSdur}={\text{QRS}}_{\text{off}}\;-\;{\text{QRS}}_{\text{on}},\;(\text{ms})$$
- 3 features for the relative QRS activity (QRSact) of the current beat, the reference template and the difference between both. QRSact is the mean QRS magnitude (mag) within 180 ms window after the QRS onset normalized to the maximal QRS magnitude. QRSact with large mean estimation is expected for the extended area QRS morphologies (VB beats).
  $$\text{QRSact}=100\frac{\overset{\text{QRSon}+180\;\text{ms}}{\underset{\text{i}=\text{QRSon}}{\text{Mean}}}({\text{mag}}_{\text{i}})}{\overset{\text{QRSon}+180\;\text{ms}}{\underset{\text{i}=\text{QRSon}}{\text{Max}}}({\text{mag}}_{\text{i}})},(\%)$$
- 3 features for the QRS mobility (QRSmob) of the current beat, the reference template and the difference between both. QRSmob feature is the ratio of the velocity area divided by the magnitude area within 180 ms window after the QRS onset that is the time domain equivalent to a higher/lower frequency content ratio. High values are expected for fast rising(falling)/small area QRS morphologies (SVB beats) and low values for slower rising(falling)/larger area QRS morphologies (VB beats). QRSmob is expected to be one of the robust integral measures but with inverse behavior compared to QRSact.
  $$\text{QRSmob}=100\frac{\sum_{\text{i}=\text{QRSon}}^{\text{QRSon}+180\;\text{ms}}\left({\text{vel}}_{\text{i}}\right)}{\sum_{\text{i}=\text{QRSon}}^{\text{QRSon}+180\;\text{ms}}\left({\text{mag}}_{\text{i}}\right)},(\%)$$
- 2 features for the current/next RR-interval durations normalized to the mean RR-interval of the last four RR-intervals:
  $$\begin{array}{l} \text{curRR}=100\cdot \frac{\text{Current RR interval}}{\text{Mean RR interval}},(\%)\\ \text{nextRR}=100\cdot \frac{\text{Next RR interval}}{\text{Mean RR interval}},(\%) \end{array}$$
- 1 feature for the relative RR-interval variability of the last 10 seconds:
  $$\text{relRRv}=100\cdot \frac{\text{Reference RR variability}}{\text{Mean RR interval}},(\%)$$

During the learning period (10s), when there is no reference template, default values are set to the reference template features: reference P-wave is present, corr = 80%, reference QRSdur = 100ms, reference QRSact = 100%, reference QRSmob = 100%.

Stage 1 extends the 20 basic features to a vector of 210 features by computing products of any two basic features (full factorial design with 2 factors). The factorial design is a common technique in exploratory statistical analyses for studying significant interactions of combinations of features (interactive second-order effects). Our goal is to produce a redundant but informative set of features which can be further optimized during the learning phase of Stage 2 classifiers.

### Stage 2: Linear-programming classifiers

#### Cluster analysis

Different approaches to clustering data have been described, divided in general at hierarchical (producing a nested series of partitions) and partitional (producing single partition) [38]. In this study, we applied the k-means partitional clustering algorithm, which is preferred for applications involving large data sets. The implemented method is shortly described:

- Step 1: Selection of k cluster centroids by either using the principle of maximal initial distance between clusters (applicable for relatively small datasets), or coinciding them with k randomly-chosen vectors in the feature space. In this study, we use random selection of the initial cluster centroids and attempt to optimize the clusterization by using the best of 10 replicates (providing maximal initial distance between clusters).
- Step 2: Assignment of each vector to the closest cluster center. In this study, the distance between vector ***x*** and the centroid of the j^th^ cluster ***z*** ^*j*^ is computed as the Euclidean distance:
  $$d_{j}=\sqrt{\sum_{i=1}^{n}{\left(x_{i}-z_{i}^{j}\right)}^{2}}\;,$$
  where *n* is the number of features.
- Step 3: Recalculation of the cluster centers using the current cluster memberships.
- Step 4: If a convergence criterion is not met, return to Step 2. Typical convergence criteria are: no (or minimal) reassignment of vectors to new cluster centers, or minimal decrease in squared error.
- Step 5: Assignment of the cluster type according to its predominant beat class membership. In this study, the generated clusters were labeled either as SVB, or as VB.

A problem accompanying the use of k-means algorithm is the choice of the number of desired output clusters. In practice, therefore, the algorithm is typically run multiple times with different starting states, and the best configuration obtained from all of the runs is used as the output clustering [38].

After generation of clusters over the training dataset, the classification of a new case is based on the type of the nearest cluster centroid (KNN classifier).

#### Fuzzy analysis

The fuzzy set theory deals with models, where the transition between full membership and no membership is gradual rather than abrupt. A fuzzy subset *A* is defined by a membership function *μ*_*A*_ *(x)*, in the range [0,1], mapping the degree to which an element *x* belongs to the fuzzy subset *A* from domain *X*.

$$\text{A}=\{(\text{x},\;\mu_{\text{A}}\;(\text{x})):\;\text{x}\in \text{X},\;\mu_{\text{A}}\;(\text{x})\in [0,1]\}$$

The general structure of a fuzzy logic classifier consists of three basic components–fuzzification unit at the input, inference block built on fuzzy logic control rules, and defuzzification unit at the output [39]. Fuzzification is a process in which by means of a membership function (triangular function, trapezoidal function, Gaussian membership function, etc.) the input features are transformed to corresponding linguistic terms to get degree of fulfillment. It means converting a crisp value into a fuzzy one by adding uncertainty. Defuzzification is the inverse process of fuzzification, in which, the output linguistic terms are converted into crisp values according to their degree of fulfillment. Widely used techniques for defuzzification are max-membership, center of gravity method, weighted average method, mean-max method and center of sums method.

This study divides the domain of all heartbeats into two fuzzy subsets belonging to SVB and VB-class. The membership functions of the crisp set of 210 features are estimated by the observed statistical distributions in SVB and VB-class, using the percentiles as a robust measure of the dispersion of the data rather than the Gaussian membership function. The confidence that a beat belongs to SVB-class or VB-class is calculated by means of the percentile ranking in the statistical distribution of the training dataset. The fuzzy logic control rule makes averaging of the confidences of the selected feature space to assign SVB or VB-class membership, according to the larger confidence value.

#### Discriminant analysis

The discriminant analysis is widely used for development of classification rules and for assessment of the relative importance of variables in the discrimination between classes [40]. In this study, a linear discriminant function is derived to separate the feature space of the two classes–SVB vs. VB. Short description of the implemented method is presented below.

Let **y**_*ij*_ be a vector of *q* features in a training dataset, in which class membership is known, for the *i* ^*-*th^ beat (*i =* 1,…, *n*_*j*_) in the *j* ^*-*th^ class (*j* = 1, 2). It is assumed that **y**_*ij*_ ~ *N*_*q*_(**μ**_*j*_, **Σ**_*j*_), where **μ**_*j*_ and **Σ**_*j*_ are the beat population mean vector and covariance matrix for the *j* ^*-*th^ class, estimated by ${\hat{\mu }}_{j}$ and ${\hat{\Sigma }}_{j}$, respectively.

The LDA classification rule assigns the *ij* ^*-*th^ vector to class 1 if:

$$\lambda (y_{ij})={\left[y_{ij}-\frac{1}{2}({\hat{\mu }}_{1}+{\hat{\mu }}_{2})\right]}^{\text{T}}\hat{a}>\text{ln}(\frac{{\hat{\pi }}_{2}}{{\hat{\pi }}_{1}})$$

Otherwise, the vector is assigned to class 2.

The symbols in the above equation are indicative for

- ^T^ is the transpose operator,
- $\hat{a}={\hat{\Sigma }}^{-1}({\hat{\mu }}_{1}-{\hat{\mu }}_{2})$is the estimate of the linear discriminant function, **a**, where:
  $$\hat{\Sigma }=\frac{(n_{1}-1){\hat{\Sigma }}_{1}+(n_{2}-1){\hat{\Sigma }}_{2}}{n_{1}+n_{2}-2}$$
- *π*_1_ and *π*_2_ are the prior probabilities that a beat belongs to class 1 or 2, respectively.

Standardized discriminant function coefficients are obtained by multiplying $\hat{a}$ with a diagonal matrix of variable standard deviations.

Considering the use of independent datasets for training and testing, as well as the large size of our training dataset (Table 1), we do not apply cross-validation or bootstrapping to calculate the accuracy of the LDA model for the training dataset. Instead, the apparent error rate is estimated during the training process, which asymptotically approximates the true prediction error for large-sized training datasets [41]:

$$APER=\frac{N-n_{11}-n_{22}}{N},$$

where *n*_11_ and *n*_22_ are the number of beats correctly assigned to class 1 and 2, respectively.

The class membership of a new beat is predicted using LDA function derived over the training dataset.

#### Classification tree

In most general terms, the purpose of the analyses via tree-building algorithms is to determine a set of ‘if-then’ logical (split) conditions that permit accurate classification or prediction of cases, known as Classification & Regression tree (C&RT) models. The target of the present study is focused on the heartbeat classification, and therefore, the presented theory is considering the C&RT model application to classification tasks.

Classification trees (CT) might be considered as a collection of rules that enable separate sets of features to be linked into a common class. The tree classifier is built by consecutive domain partitions of the feature set until uniformity is attained in created subsets. The tree resembles a graph that consists of a root node from which at least two branches emerge, which then lead to inferior nodes (child nodes). Each node is attributed to a class description, and each branch refers to a decision rule, i.e. a condition related to features from the input data set and describing the case when each branch is chosen. The most popular type of a decision rule is the so called univariate split based on testing a single feature. By successive splitting of the dataset, the child nodes become parent nodes. In common algorithms, the conditions on the branches of each node must be complementary in a manner that provides one possible path downward when ‘climbing the tree’. Nodes that do not have any child nodes are known as leaf, terminal nodes or final decision nodes, and represent the final classes.

Tree design approaches are aimed at finding ‘optimal’ solutions: minimum sized trees with high classification accuracy. Since a search on the whole set of possible trees for a given problem is almost always impractical, this study follows the most common tree building strategy with two steps [42]:

- Step 1: Splitting of nodes–growing a tree, in a top-down way, until all possible leaf nodes have been reached (i.e. purity), based on specific splitting criteria. The most popular concept is based on splitting of independent variables at several split points taking into account the homogeneity of data at each node. Rigorous measures of impurity, based on computing the proportion of the data that belong to a class, such as entropy (maximal deviance reduction), Gini index, twoing rule are among the most commonly used splitting criteria to quantify the homogeneity in CT.
- Step 2: Pruning the tree by backward removing of particular branches based on specific pruning criteria, remedying the usual over-fitting of the final solution reached by Step 1. Complex trees usually exhibit meaningless extra nodes, i.e. with decision rules that don’t make sense in terms of medical knowledge, and therefore, the accuracy, efficiency and generalization capability of the final solution relies on using an optimal scheme for tree pruning. To guide the tree pruning, the misclassification rate is typically measured so that branches giving less improvement in error cost are first pruned.

The mathematical background presented below is defining the most common criteria for splitting nodes and pruning the tree, which are the basis for the CT model development with special application to heartbeat classification. The criteria are formulated for general number of classes, which can be simplified to a two class problem, considering the target heartbeat assignment to SVB and VB-classes.

Let assume that there are *n* observations in a parent node *P* and there are *J* classes labeled as *1*,*2*,*3*,*…J*. Let *n*_*j*_ be the number of beats in class *j*. The relative proportion *n*_*j*_/*n* of class *j* beats in the node is denoted by *p*_*j*_. Each binary split *s*_*i*_ produces two child nodes–left (*L*), which contains *n*_*L*_ beats and right (*R*) with *n*_*R*_ beats, such that *n*_*L*_ *+n*_*R*_ *= n*. The child nodes contain the relative proportions *p*_*L*_ *= n*_*L*_ */n* and *p*_*R*_ *= n*_*R*_ */n*. The relative proportions of class *j* beats in the child nodes are denoted by *p*_*jL*_ and *p*_*jR*_. The notation *i(p)* is further used as a generic notation of impurity, formulated below for the three most common splitting methods.

### *-* Splitting based on entropy or maximal deviance reduction

The entropy is defined as:

$$i_{E}(p)=-\sum_{j=1}^{J}p_{j}\;\text{log}\;p_{j}$$

When the entropy of a node is zero, *p*_*j*_ = 1 for class j, then the node is said to be pure, since it contains beats of only one class. When the entropy is maximized, *p*_*j*_ is uniform, then the node is least pure because it contains equal proportions of beats from each class.

It is shown that the entropy and deviance based measurements of impurity differ by a constant factor [43]. The deviance is defined as:

$$i_{D}=-2\sum_{j=1}^{J}n_{j}\;\text{log}\;p_{j}=2ni_{E}(p)$$

In our case with two classes, this formulation can be reduced to:

$$i_{D}(p)=2n(-p_{1}\;\text{log}\;p_{1}-p_{2}\;\text{log}\;p_{2})=2n(-p_{1}\;\text{log}\;p_{1}-(1-p_{1})\;\text{log}(1-p_{1}))$$

These methods are valuable for splitting leaves that contain a large number of observations [44].

### - Splitting based on Gini index

The Gini index is defined as:

$$i_{G}(p)=\sum_{p_{j}\neq p_{i}}p_{i}p_{j}=1-\sum_{j=1}^{J}p_{j}^{2}$$

In our two-class problem, the Gini index can be simplified to:

$$i_{G}(p)=1-(p_{1}^{2}+p_{2}^{2})=1-p_{1}^{2}-{\left(1-p_{1}\right)}^{2}=2p_{1}(1-p_{1})$$

This method looks for the largest class in a dataset and tries to isolate it from the other classes. The Gini index is equivalent to second-order entropy [45] and is claimed to result in trees that are structurally similar to those, obtained when entropy (deviance) is used for splitting [46,47].

The goal of the maximum deviance (entropy) reduction and Gini index is to reduce the uncertainty until a pure leaf node is established.

### - Splitting based on twoing rule

The twoing rule [46] has a much different splitting strategy than maximum deviance (entropy) reduction and Gini index, and tends to be most useful in multi-class tree creation. It searches for the split that maximizes:

$$i_{T}(p)=\frac{p_{L}p_{R}}{4}{\left(\sum_{j=1}^{J}\left|p_{jL}-p_{jR}\right|\right)}^{2}$$

This rule aims to divide the classes between the child nodes in a way that equal size nodes are formed.

### - Pruning based on misclassification rate

The misclassification rate is an error function, which measures the discrepancy between the beat annotations and the model’s outputs (i.e. the cost of misclassifying a beat from class VB as belonging to class SVB and vice versa).

Considering a node *m*, the proportion of a class *j* at this node is defined as:

$$p_{jm}=\frac{1}{n_{m}}\sum_{k=1}^{n_{m}}I(y_{k}=j),j=1,2,3\ldots J\;,$$

where *n*_*m*_ is the number of beats in the tree node *m*, *y*_*k*_ is the tree response for beat *k*, and *I* is an indicator function.

The observations in node *m* are classified to the majority class:

$$j(m)=\text{arg max}[p_{jm}]$$

The misclassification error applied to guide the cost-complexity pruning of the tree is defined as:

$$MCE=\frac{1}{n_{m}}\sum_{k=1}^{n_{m}}I(y_{k}\neq j(m))=1-\text{max}\left\{p_{jm}\right\}$$

Pruning a node and its descendants leads to misclassification error increase, so that the pruning algorithm iteratively selects to first prune the node with minimal influence on the misclassification rate.

**Fig 2:**
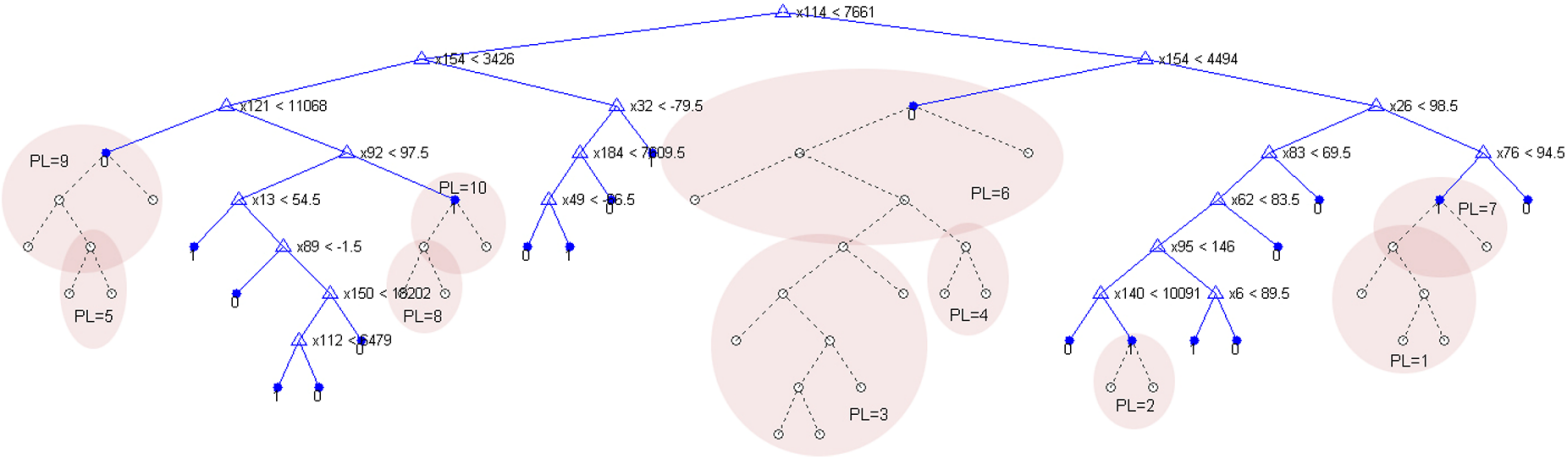
General view of an example CT model. CT has a total number of 73 nodes, 36 branches, 20 decision nodes, including 31 variables (identification numbers are assigned as x114, x154, x121, etc. counting the input vector of 210 features). The classification process starts at the root of the tree. An incoming beat travels down the branches of the tree depending on the result of the test on a feature. The procedure ends when the beat arrives at a leaf ‘1’ (SVB-class) or ‘0’ (VB-class). The tree is backwards pruned to level 10, and the pruned nodes and branches are shown with dotted lines. The subsequently pruned groups of nodes and branches are highlighted in common areas, starting from pruning level PL = 1, 2, …10.

Fig 2 shows a simple CT model, which is first split to a number of 20 final decision nodes, and then it is backwards simplified to different pruning levels.

The CT model exhibits some important benefits:

- No need for prior optimization of the input feature space: The self-learning algorithm analyses the training set and grows the tree in a stepwise mode by entering the feature which has the most significant contribution for minimizing the error cost function.
- The final CT model can be easily interpreted as a set of ‘if-then’ rules. Such algorithm is fast executable and applicable in real-time environment;
- The geometry of CT is simple and comes with a visual interpretation of the conjugation of tests (the path leading from the root node to the final decision node);
- CT explicitly localizes the ranges of the feature space that are pertinent to the given class of beats;
- Derivation of results without a need for deep knowledge on the tested beat features that increases the practical applications of CT for solving heartbeat classification problems, suggested also for more than two classes.

## Results

The heartbeat classification performance of Stage 1 and Stage 2 are estimated by three statistical indices that are adopted in the research community to provide comprehensive assessment of imbalanced learning problems [48]: sensitivity (Se), specificity (Sp), positive predictive value (PPV).

$$Sp=\frac{TN}{TN+FP}\;,\;Se=\frac{TP}{TP+FN}\;,\;PPV=\frac{TP}{TP+FP}\;,$$

where TN and FP are true negatives and false positives for SVB-class; TP and FN are true positives and false negatives for VB-class.

**Fig 3:**
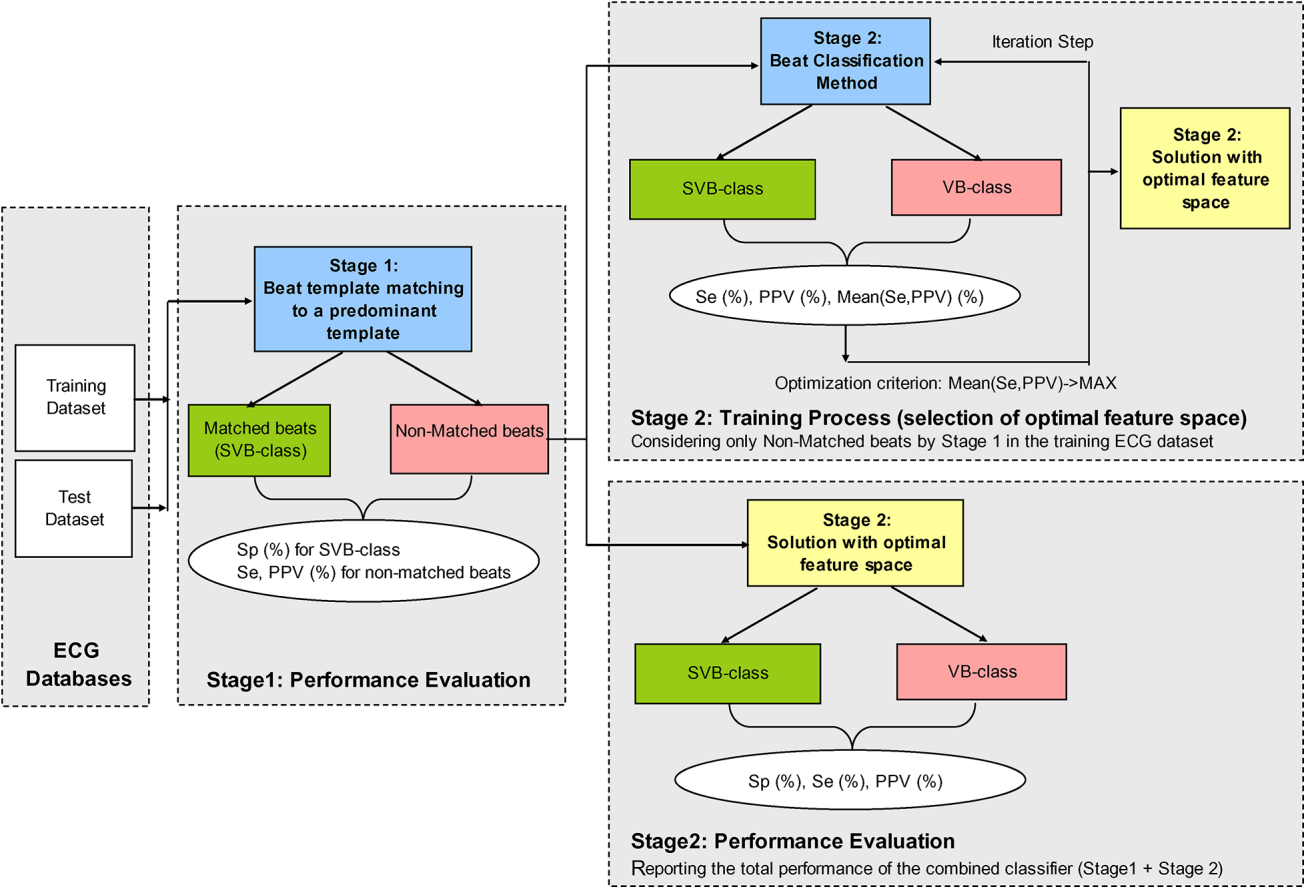
Block-diagram of the training and test-validation of the two-stage beat classifier. It shows the performance evaluation of Stage 1, the training process of Stage 2 and the performance evaluation of the combined classifier (Stage 1+Stage 2).

The scheme for performance evaluation of Stage 1, the training process of Stage 2 and the performance evaluation of the combined classifier (Stage 1+Stage 2) is presented in Fig 3.

### Stage 1: Performance evaluation

Stage 1 performance is evaluated by the statistical indices Se, Sp, PPV when considering the following interpretation of TN, FP, TP, FN cases:

- For N and S beats (SVB-class):
  ○ TN = Matched beats to the predominant beat template
  ○ FP = Non-matched beats to the predominant beat template
- For V and F beats (VB-class):
  ○ TP = Non-matched beats to the predominant beat template
  ○ FN = Matched beats to the predominant beat template

The performance of Stage 1 for the training and test databases is presented in Table 2. The considerably low value of PPV, achieved at the output of Stage 1 (about 36%), indicates for the need of a second beat classification stage dedicated to decrease of the FP rate.

**Table 2:** Classification performance of Stage 1.

Stage 1 decision	Matched beats to a predominant template (assigned to SVB-class)	Non-matched beatsto a predominant template	
	Sp (%)	Se (%)	PPV (%)
**Training databases**			
EDB	95.86	96.20	12.48
SVDB	93.09	92.76	43.39
AHA	94.79	93.97	66.05
**Total training**	**95.28**	**93.93**	**36.29**
**Test database**			
MIT-BIH	**91.22**	**94.39**	**48.05**

### Stage 2: Training process

All beat classification methods designed in Stage 2 (including Cluster, Fuzzy, LDA and CT models) are implemented in Matlab7.5 (The Mathworks Inc.), using the standard functions embedded in the Statistical toolbox. The input dataset for the training process of Stage 2 involves those beats from the training databases (EDB, AHA, SVDB) which are not matched to the predominant beat template by Stage 1, i.e. 50175 N-beats, 2757 S-beats, 29263 V-beats, 900 F-beats. The training is an iterative process for selection of the optimal feature space, trying at each step to minimize the number of errors. We consider at equal weight the FPs in SVB-class and FNs in VB-class, therefore, the optimization criterion aims to maximize Se and PPV, considered to effectively evaluate the classification performance in imbalanced learning scenarios [48], by the common index:

$$Mean(Se,PPV)=\frac{\left(Se+PPV\right)}{2}.$$

#### Cluster analysis

The cluster model implemented in the study has two settings influencing the output–the number of clusters and the selected feature space. These items are not independent, but rather the selection of the feature space affects the cluster number and vice versa. The maximal number of clusters is limited to $\sqrt{210features/2}\approx 10$ clusters following the ‘rule of thumb’ [49]. The training process involves a stepwise cluster analysis with a fixed number of clusters (2, 3, … 10), applying iterative selection of the optimal feature space of the model as follows:

First iteration step:

○ S1: Selection of one feature from the input feature space.
○ S2: Clusterization with 10 replications of the initial cluster centroid positions. The best replication is estimated by accuracy: [Se, Mean(Se,PPV)] for the training dataset.
○ S3: All features are one-by-one subjected to S2 for final selection of the best-performing feature, providing maximum of Mean(Se,PPV). If several features have equal values of Mean(Se,PPV), the one with the highest Se is chosen.
  - Next iteration steps:
○ S4: Selection of a feature which is still not included in the model and adding it to the feature space selected at the previous step. The new feature space is subjected to S2.
○ S5: All relevant features are one-by-one subjected to S4 and the best performing solution at this iteration step is selected according to the criteria in S3.
  - The optimal iteration step: it is defined when cluster analysis stops to improve performance, e.g. any new feature in the feature space at any further step does not produce increased values of Mean(Se,PPV) nor Se.

**Fig 4:**
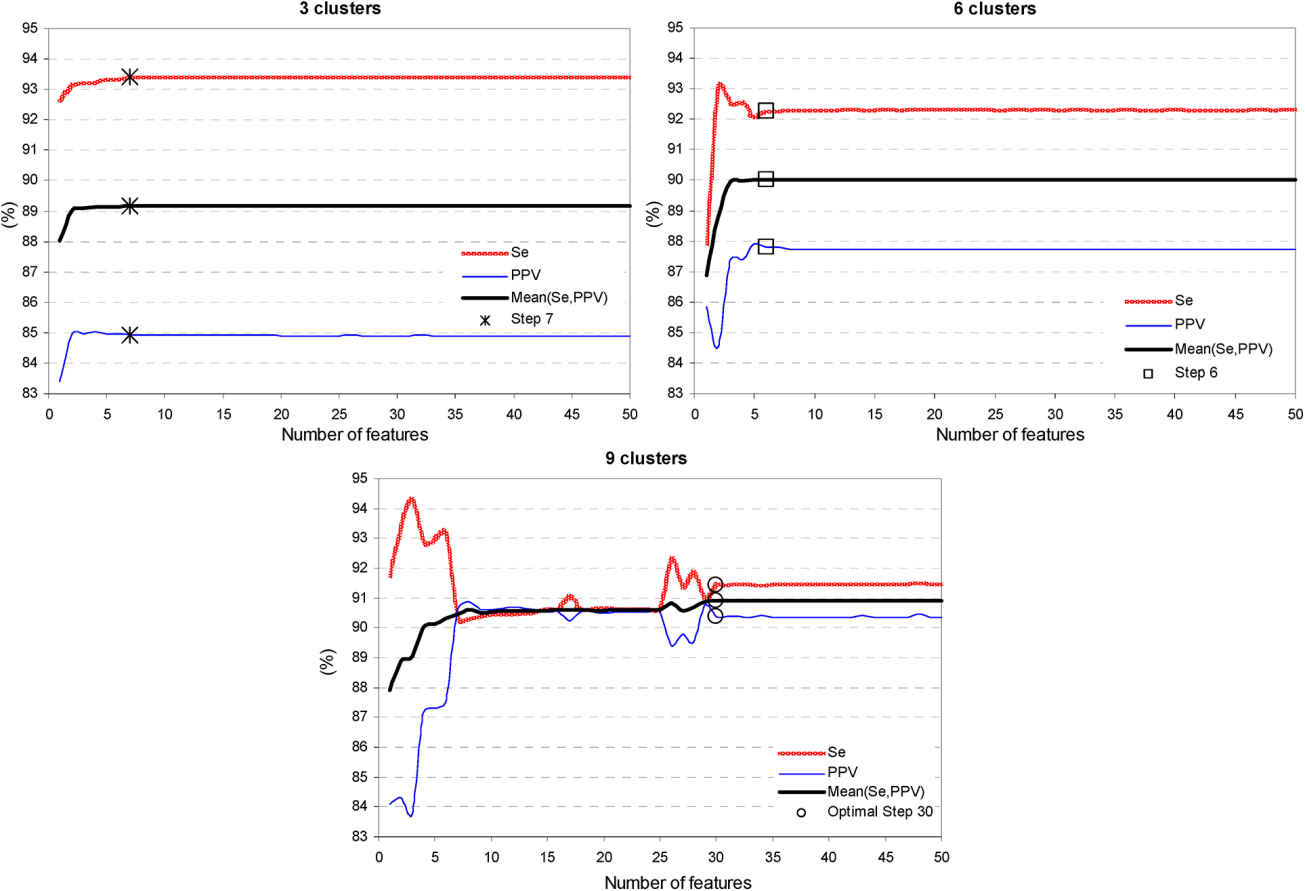
Training process of the stepwise cluster analysis: influence of the number of features entered in the model. The trend of Se, PPV, Mean(Se,PPV) is reported for 3, 6, 9 clusters. The optimal iteration step is defined for 7 features (3 clusters, ‘*’ mark), 6 features (6 clusters, ‘□’ mark) and 30 features (9 clusters, ‘○’ mark).

**Fig 5:**
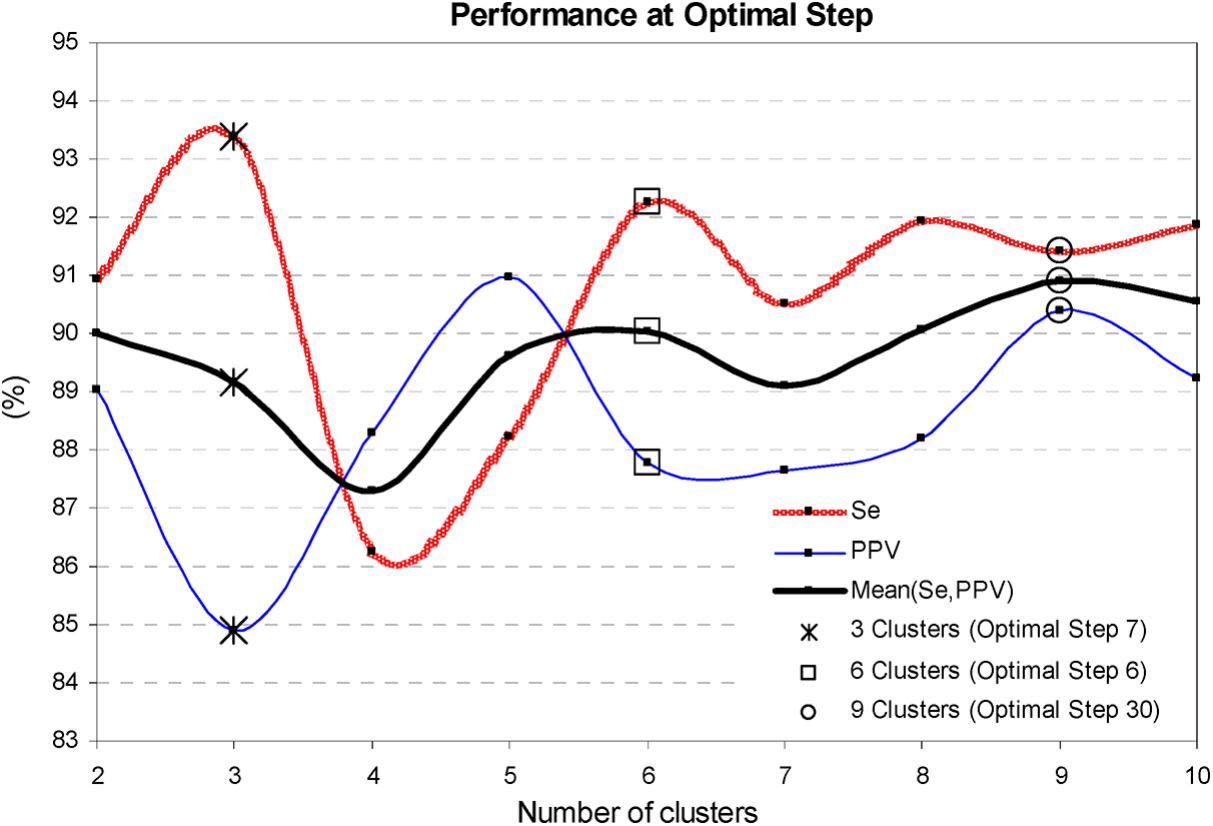
Training process of the stepwise cluster analysis: influence of the number of clusters. The values of Se, PPV, Mean(Se,PPV) are reported at the optimal iteration step (3, 6, 9 clusters are assigned with the same marks as in Fig 4). The best performing solution is defined for 9 clusters (Step 30). The continuous performance graphs are depicted by spline interpolation between the solutions at integer number of clusters.

The training process of the stepwise cluster analysis is illustrated in Figs 4 and 5. Fig 4 is an evidence that there could be clearly defined a minimal feature space when the cluster’s performance reaches saturation, e.g. the optimal iteration step for 3 clusters involves 7 features, for 6 clusters– 6 features, for 9 clusters– 30 features. Fig 5 presents the cluster’s best performing solutions which fluctuate within 5% when the number of clusters is set from 2 to 10 clusters. We observe the worst performance (87.2%, 86.3%) for 4 clusters, and the best performance (90.9%, 91.4%) for 9 clusters, reported as (Mean(Se,PPV), Se). Therefore, we define the use of 9 clusters (5 assigned to SVB-class, 4 assigned to VB-class) and 30 features as the best performing cluster analysis solution in Stage 2.

#### Fuzzy analysis

The accuracy of the fuzzy analysis depends on the features involved in the model, i.e. on their average confidence that a beat belongs to SVB or VB-class. The training applies a stepwise fuzzy analysis with iterative selection of the optimal feature space, implying a larger difference between both confidences. The training process follows the same iteration steps (S1 to S5) as defined for the cluster analysis. The only difference is the model specific estimations at step S2, where the fuzzy analysis calculates the average fuzzy distribution confidence of the selected feature space. At the optimal iteration step, the fuzzy analysis stops to improve performance, e.g. any new feature in the fuzzy model at any further step does not produce increased values of neither Mean(Se,PPV), nor Se.

**Fig 6:**
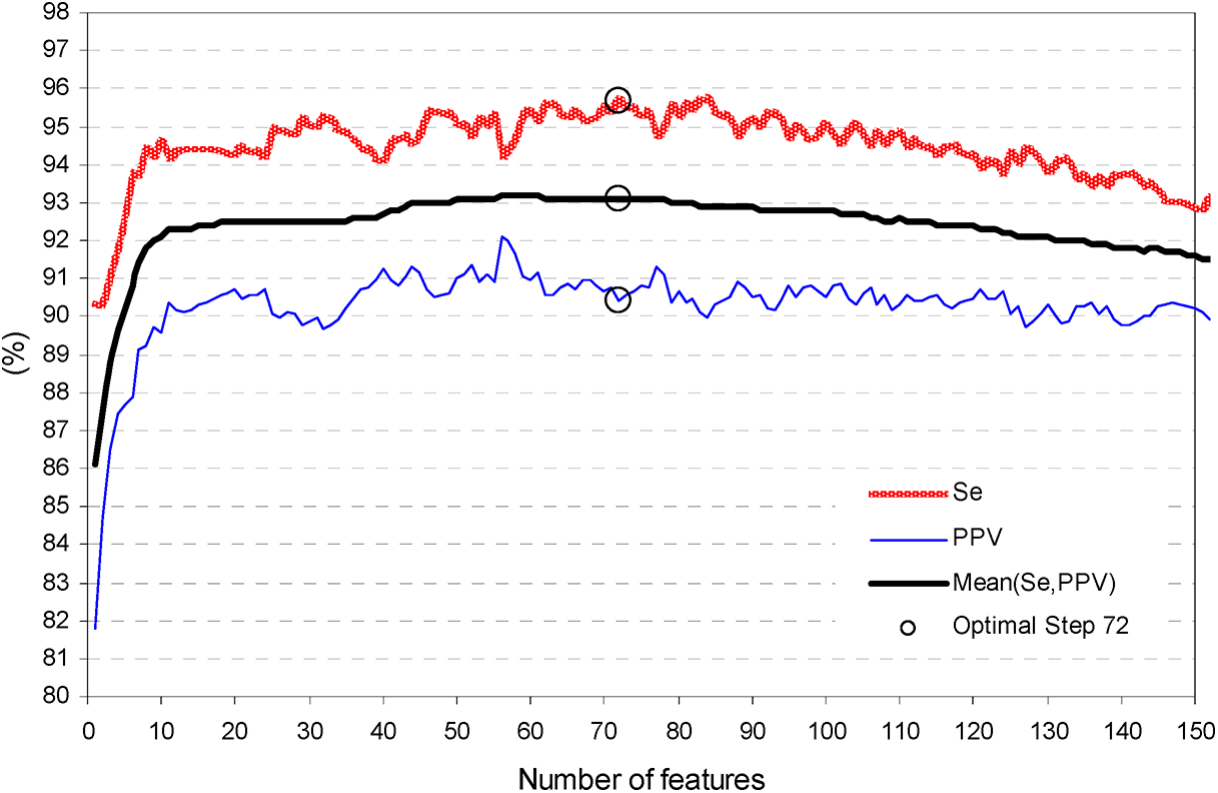
Training process of stepwise fuzzy analysis: influence of the number of features entered in the model on the trend of Se, PPV, Mean(Se,PPV). The optimal iteration step is defined for 72 features at maximal Se during a plateau of Mean(Se,PPV)–see (‘○’ mark).

Fig 6 illustrates the training process of the fuzzy analysis, which presents an increase of Mean(Se,PPV) by 7% (from 86.1 to 93.1%) and Se by 5.3% (from 90.4 to 95.7%) when a single feature fuzzy model is extended to the optimal set of 72 features. Further addition of features is deteriorating both Mean(Se,PPV) and Se.

#### Discriminant analysis

The LDA model implemented in this study has two settings which affect the output–the prior probability for SVB vs. VB-class and the selected feature space. Varying the prior probability of one class scales the constant of the discriminant function so that the feature space is divided in advantage to this class. Different prior probabilities for SVB vs. VB-class (i.e. from 70/30% to 10/90%) were tested during the training process, thus varying the ratio between Se and PPV.

Stepwise LDA (SDA) is applied to combine features in order to obtain a linear discriminant function, which best separates the feature space of SVB vs. VB-class. SDA is iteratively trained as follows:

- First iteration step: All features are one-by-one involved in independent LDA functions and the feature with the best discrimination ability (the highest Mean(Se,PPV)) over the training database is selected.
- Next iteration steps:
  ○ New feature is included in the discriminant function of the previous step. The new feature is chosen among the set of all features, which are still not included in the SDA model.
  ○ If in a specific step, some entered feature is correlated to any of the features already in the model, and produces badly scaled pooled covariance matrix, it is excluded from the current and next iteration steps.
  ○ The selection process iteratively makes assessment of the Mean(Se,PPV) for each new feature in the model and finally selects the feature, which provides maximal value of Mean(Se,PPV). If several features have equal values of Mean(Se,PPV), the one with the highest Se is chosen.
- The optimal iteration step: it is defined when SDA stops to improve performance, e.g. any new feature in the discriminant function at any further step does not produce increased values of Mean(Se,PPV) nor Se. It is important to stop at the earliest step of best SDA performance, thus providing the minimal complexity of SDA (i.e. minimal number of discriminant function coefficients equal to the iteration step number).

**Fig 7:**
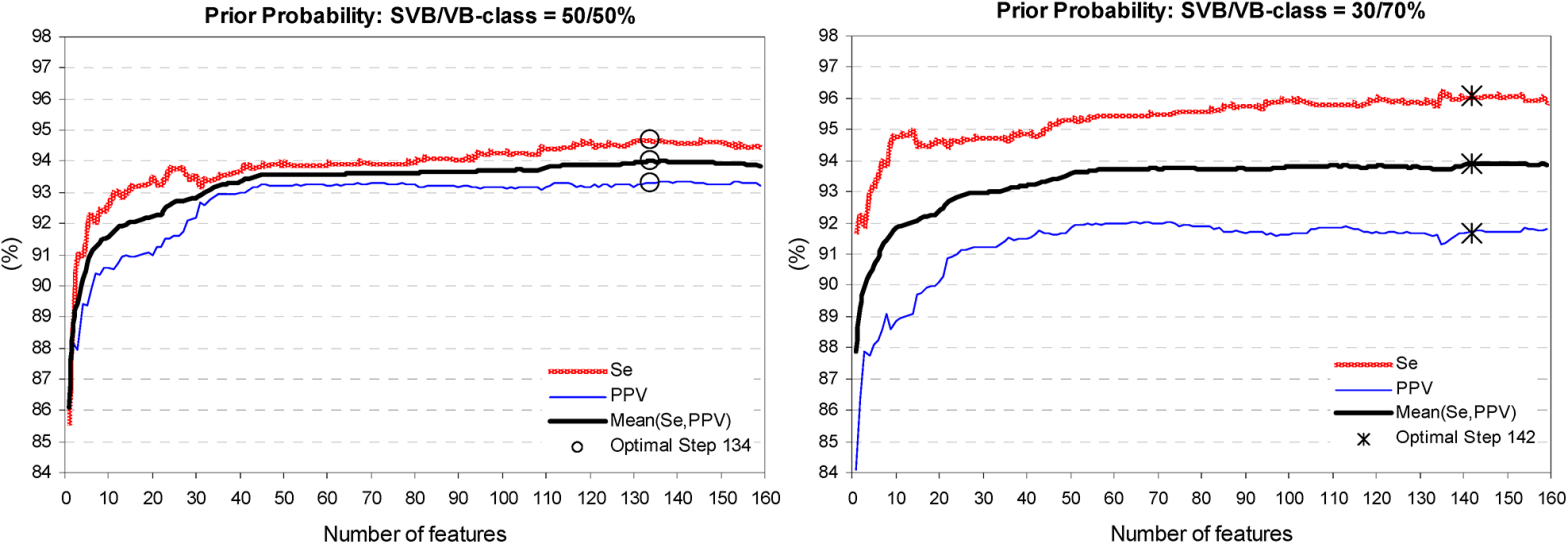
Training process of SDA: influence of the number of features entered in the model. The trend of Se, PPV, Mean(Se,PPV) is reported for different settings of the prior probabilities of SVB vs. VB-class: 50%/50% and 70/30%. The optimal iteration step is defined for 134 features (50%/50%, ‘○’ mark) and 142 features (30%/70%, ‘*’ mark).

**Fig 8:**
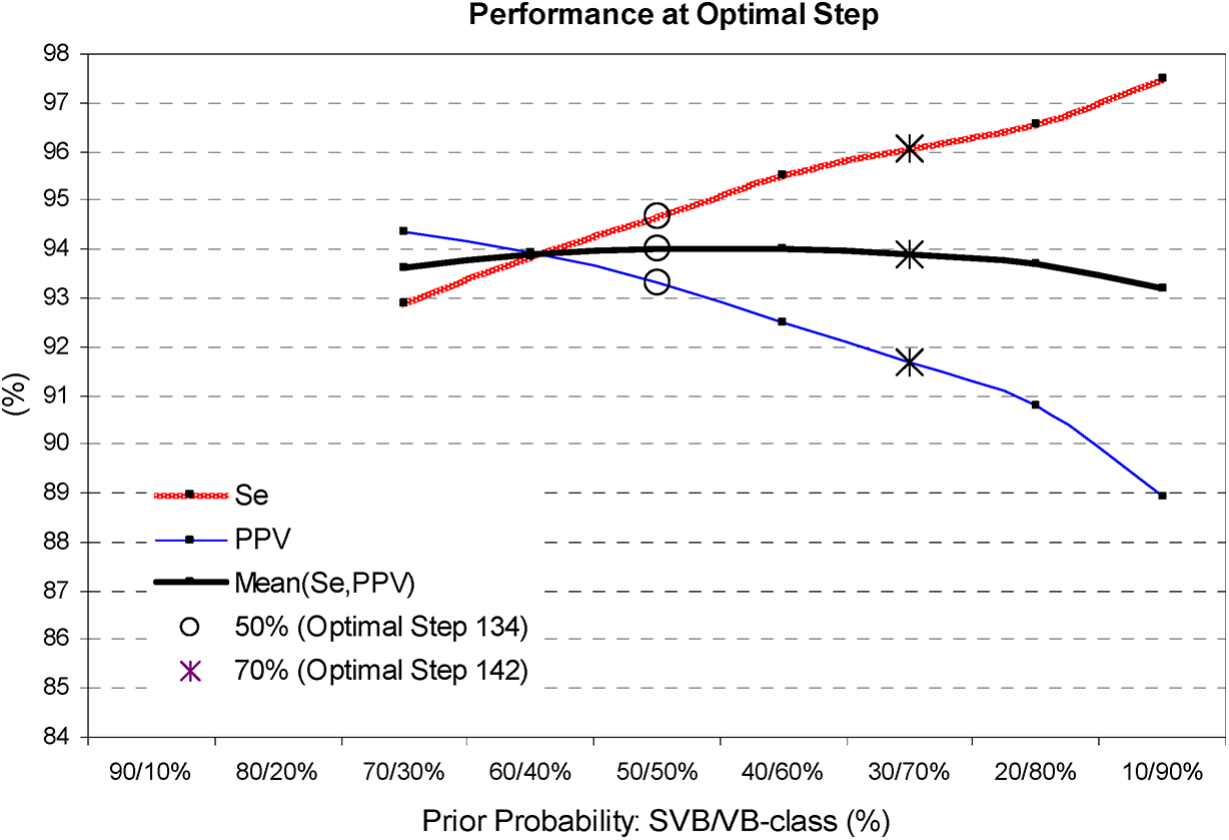
Training process of SDA: influence of the prior probability of SVB vs. VB-class. The values of Se, PPV, MeanSePPV are reported at the optimal iteration step (50%/50% and 30%/70% are highlighted by the same marks as in Fig 7). The best performing solution is defined for prior probability of 30%/70% (Step 142).

Fig 7 illustrates the training process of SDA, when the performance is improved by each new feature entered in the model. A single feature LDA model has Mean(Se,PPV) as low as 86–88%, which is improved to 94% by the optimal feature set selected at 134 and 142 iteration step, considering SVB/VB-class prior probabilities of 50%/50% and 30%/70%, respectively. Fig 8 presents the best SDA performance when varying the SVB/VB-class prior probability from 70%/30% to 10%/90%. It is evident that increasing the VB-class prior probability from 30% to 90% increases Se by 4.5% points (from 93% to 97.5%) but PPV is proportionally decreasing (from 94.5% down to 89%). The optimal prior probability is considered the one with Se>PPV but not deteriorated Mean(Se,PPV), that is seen for 30%/70% (Mean(Se,PPV) = 94%, Se = 96.1%). Therefore, we define the discriminant function with 142 features achieved for SVB/VB-class prior probability of 30%/70% as the best performing LDA classifier in Stage 2.

#### Classification Tree

**Fig 9:**
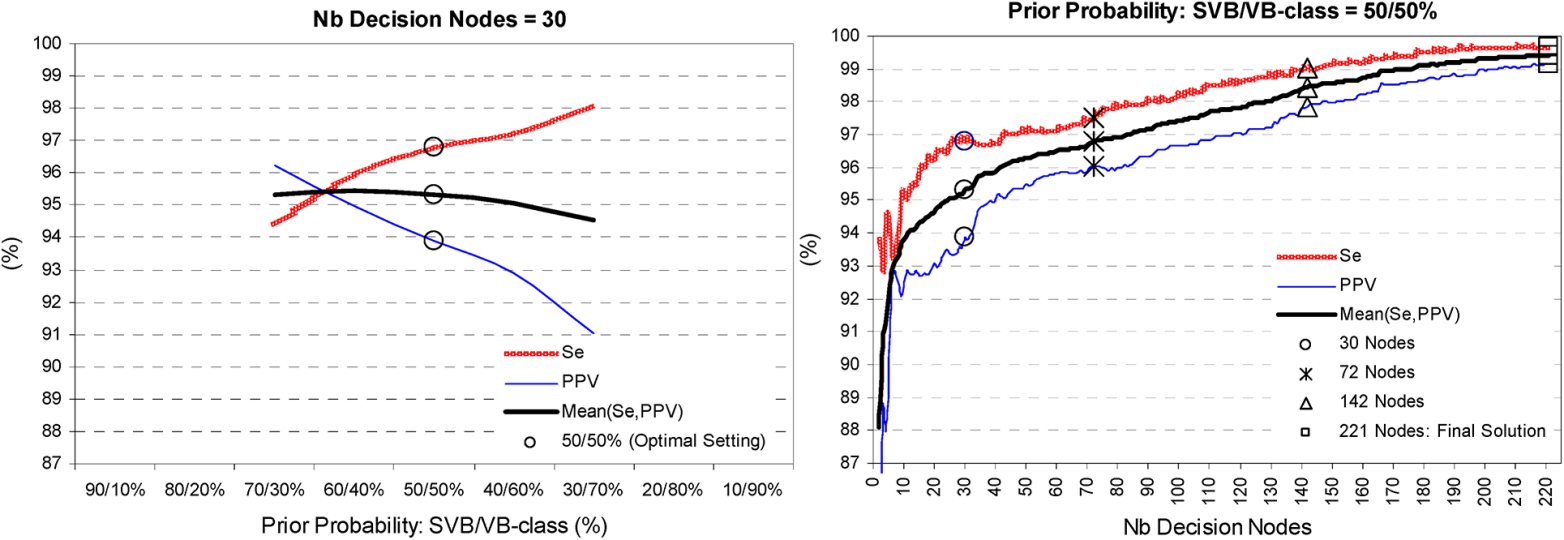
Training process of CT. It shows the trend of performance: Se, PPV, Mean(Se,PPV) in respect of:—left graph: different prior probabilities of SVB vs. VB-class. The optimal setting is defined for equal prior probabilities 50%/50% (‘o’ mark at maximal Se over the Mean(Se,PPV) plateau).—right graph: the number of final decision nodes after pruning of the tree. The solutions with 30, 72, 142 nodes are highlighted (Mean(Se,PPV) = 95.3%, 96.8%, 98.5%; Se = 96.9%, 97.4%, 99%) as they correspond to the number of features in the best performing Cluster, Fuzzy, LDA models, respectively. The maximal CT performance at the final splitting step is also marked–Mean(Se,PPV) = 99.4%, Se = 99.7%.

**Fig 10:**
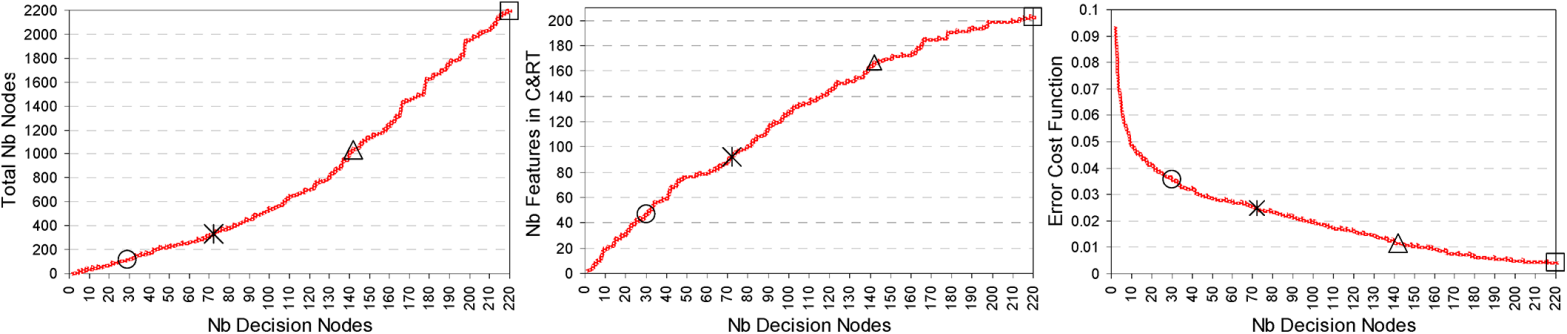
Complexity of the CT model evaluated in Fig 9 (right graph). It shows the relationship between the number of the decision nodes vs. (i) the total number of nodes, (ii) the number of features included in the model, (iii) the error cost function, all marked for the highlighted solutions with 30, 72, 142 and 221 decision nodes.

In this study, the CT model is generated and pruned by means of the statistical Matlab toolbox, using the following settings:

- Two categories of the classification variable according to the beat annotation: SVB or VB class.
- Splitting criterion set to ‘maximum deviance reduction’, which is advantageous in our case of large sample size. The splitting of the decision tree is a self-learning algorithm, which analyzes the training set to develop the most successful strategy for growing a tree structure with the highest performance. The growing of the tree is terminated when a stop condition of minimum size of impure node to be split is reached (the setting is 10). The maximum splitting level of the best performaning CT model in Fig 9 (right graph) contains 221 decision nodes.
- Different prior probabilities of SVB vs. VB-class (i.e. 70%/30%, 60%/40%, 50%/50%, 40%/60%, 30%/70%)–the prior probabilities affects the relative proportions of both classes involved in the node impurity measurements during the splitting process, thus varying the ratio between Se and PPV. The CT training process aims to define the optimal prior probability setting, which attains the highest Se and Mean(Se,PPV), that is proved for 50%/50% in Fig 9 (left graph).

- Pruning criterion based on misclassification rate–A subsequent pruning of the best performing CT model with 221 decision nodes is applied in order to simplify the decision rules which overfit the data. The training process scans the whole range of pruning levels, i.e. the CT model is backwards simplified from 221 to 2 decision nodes in order to evaluate the relationship between the complexity of the tree vs. its sensitivity and positive predictivity (Fig 9 –right graph) and additionally the complexity of the CT model itself as a total number of nodes, number of features and error cost function (Fig 10). Considering an acceptable pruning level with error cost <0.05, seen for more than 10 decision nodes in our CT model (Fig 10 –right graph), the CT solutions which are selected for validation in Stage 2 classifier include 30, 72, 142, 221 decision nodes, the first three corresponding to the number of iteration steps used to build the best performing Cluster, Fuzzy and LDA models–highlighted in Figs 9 and 10.

### Stage 1 + Stage 2: Total performance evaluation

The performance of the combined classifier (Stage 1 + Stage 2) is evaluated by the statistical indices Se, Sp, PPV when considering the following interpretation of TN, FP, TP, FN cases:

- For beats annotated in the databases as N and S (SVB-class):
  ○ TN = Matched beats to predominant beat template (Stage 1) + Beats assigned to SVB class (Stage 2)
  ○ FP = Beats assigned to VB class (Stage 2)
- For beats annotated in the databases as V and F (VB-class):
  ○ TP = Beats assigned to VB class (Stage 2)
  ○ FN = Matched beats to predominant beat template (Stage 1) + Beats assigned to SVB class (Stage 2)

Comparative study of the combined classifier when Stage 2 implements the best performing solutions of 4 heartbeat classification methods is presented in Table 3, reporting the independent test-validation on MIT-BIH database. The values of Se and PPV are calculated for the subgroup of V-beats (part of VB-class annotated in the databases as V beats, excluding F beats) seeking for comparison to the usual performance reports of other published heartbeat classifiers (as shown in Discussion).

**Table 3:** Test performance of the combined beat classifier (Stage 1 + Stage 2) on MIT-BIH database. Different Stage 2 classification methods are compared–The maximal performance of stepwise Cluster (step 30), Fuzzy (step 72), LDA (step 142) models with optimal number of steps vs. CT models with an equivalent number of final nodes. The maximal accuracy of the CT final solution (221 nodes) is also reported. Sp is calculated for SVB-class; Se, PPV are calculated for VB-class, as well as only for V-beats (part of VB-class), the latter performance being usually reported in other published heartbeat classifiers.

	SVB-class	VB-class			
	(N+S beats)	(V+F beats)		(V beats)	
	Sp (%)	Se (%)	PPV (%)	Se (%)	PPV (%)
Cluster (step 30)	99.5	87.4	93.9	92.4	93.6
CT (nodes 30)	99.6	91.1	95.3	94.7	95.0
Fuzzy (step 72)	99.4	90.4	92.8	94.4	92.4
CT (nodes 72)	99.7	92.4	96.5	95.5	96.2
LDA (step 142)	99.5	90.6	93.4	94.2	93.0
CT (nodes 142)	99.8	93.4	98.1	96.3	97.9
CT (nodes 221 –Final solution)	99.9	94.1	99.3	96.7	99.2

## Evaluation of the Features

An overview of the 20 basic features and their potential for providing separable distributions for SVB vs. VB-class is estimated for all beats in the training dataset, input to Stage 2 (see Table 4). Comparing SVB vs. VB-class, we can highlight specific continuous features among the set from F6 to F20 in Table 4, giving the least overlapping distributions (mean±std) with grounded physiological meaning:

- F6: the mean beat correlation to the reference template is about 30% points smaller in VB-class;
- F11: the mean QRS duration (beat-to-template difference) is about 40 ms larger in VB-class;
- F12, F14: the mean QRS activity (a measure of the QRS area) of the beat and the beat-to-template difference are about 50% points larger in VB-class;
- F15, F17: the mean QRS mobility (a time-domain measure of higher/lower frequency content ratio) of the beat and the beat-to-template difference are about 30% points smaller in VB-class;
- F18: the mean current RR-interval duration is about 30% points shortened in VB-class.

The most outstanding discrete features (F1 to F5) are F2 and F3: the frequency of observation for the template types matched by previous and next beats are 1.5 to 3 times different.

The extended set of 210 features is introduced to enhance the potential second-order interactions between any two basic features. Although this is a redundant set, we provide all features in parallel to the input of the Stage 2 classifier so that it is able to iteratively select the optimal feature, which best improves its performance on a specific learning step. We give below the top-10 ranked features’ second-order interactions, which are automatically selected by the specific Stage 2 classifier in its optimal feature space during the first 10 learning steps:

- **Cluster**: F6*F18, F8*F19, F15*F18, F15*F11, F15*F17, F18*F19, F6*F17, F10, F12*F20, F20*F11;
- **Fuzzy**: F6*F18, F15*F18, F5*F11, F18, F4*F6, F5*F7, F6*F14, F5*F8, F1*F16, F13*F16;
- **LDA**: F6*F18, F7*F14, F1*F5, F15, F10*F16, F1*F2, F4*F12, F13*F18, F12*F15, F1*F4;
- **CT**: F6*F18, F9*F14, F7*F9, F1*F7, F1*F15, F15*F18, F5*F7, F15, F4*F6, F9*F18.

**Table 4:** Statistical distribution of the basic features for SVB and VB-class evaluated for the beats in the training dataset that are supplied to the input of Stage 2. The discrete features (F1-F5) are reported as frequency of observation; the continuous features (F6-F20) are represented as Mean±Std. The top-10 ranked second-order feature interactions selected by Cluster, Fuzzy, LDA, CT models are denoted in the row of each involved feature, specifying the index of the coupling feature in the interaction. If one feature is involved in several interactions, then the order of their selection in the model is used to list the respective coupling features.

				Contribution to the top 10-ranked second-order interactions			
Feat.	Description	SVB-class	VB-class	Cluster	Fuzzy	LDA	CT
**F1**	Template matched by current beat			-	F16	F5	F7
	- reference:	0% *	0% *			F2	F15
	- other:	68.6%	71.3%			F4	
	- none:	31.4%	28.7%				
**F2**	Template matched by prev. beat			-	-	F1	-
	- reference:	48.0%	82.3%				
	- other:	31.1%	10.9%				
	- none:	20.9%	6.8%				
**F3**	Template matched by next beat			-	-	-	-
	- reference:	48.9%	81.4%				
	- other:	31.1%	10.8%				
	- none:	20.0%	7.8%				
**F4**	P-wave present in current beat	87.4%	62.0%	-	F6	F12	F6
						F1	
**F5**	P-wave present in ref. template	90.2%	94.9%	-	F11	F1	F7
					F7		
					F8		
**F6**	corr of current beat	89.4±12.0%	60.6±24.9%	F18	F18	F18	F18
				F17	F4		F4
					F14		
**F7**	corr of previous beat	91.6±12.5%	93.3±15.8%	-	F5	F14	F9
							F1
							F5
**F8**	corr of next beat	91.8±12.2%	93.5±14.5%	F19	F5	-	-
**F9**	QRSdur of current beat	138.6±50.0 ms	184.1±38.4 ms	-	-	-	F14
							F7
							F18
**F10**	QRSdur of reference template	122.7±32.2 ms	126.4±29.3 ms	F10	-	F16	-
**F11**	QRSdur beat-template difference	15.9±44.7 ms	57.7±39.4 ms	F15	F5	-	-
				F20			
**F12**	QRSact of current beat	124.8±35.9%	172.0±34.7%	F20	-	F4	-
						F15	
**F13**	QRSact of reference template	113.9±32.9%	109.9±29.2%	-	F16	F18	-
**F14**	QRSact beat-template difference	10.9±33.7%	62.1±38.4%	-	F6	F7	F9
**F15**	QRSmob of current beat	90.8±22.4%	60.0±19.0%	F18	F18	F15	F1
				F11		F12	F18
				F17			F15
**F16**	QRSmob of reference template	93.4±19.6%	92.5±18.6%	-	F1	F10	-
					F13		
**F17**	QRSmob beat-template difference	-2.6±18.4%	-32.5±22.3%	F15	-	-	-
				F6			
**F18**	curRR	100.1±12.8%	73.1±16.4%	F6	F6	F6	F6
				F15	F15	F13	F15
				F19	F18		F9
**F19**	nextRR	100.0±12.0%	121.5±23.1%	F8	-	-	-
				F18			
**F20**	relRRv	4.7±5.6%	5.7±7.7%	F12	-	-	-
				F11			

Note:*All heartbeats that matched the reference template are assigned to SVB-class by Stage1.

The top-10 ranked features give an insight to the principal second-order interactions (denoted in Table 4). We remark that all 4 models select at the first step the same feature interaction (F6*F18) that is a sign for a robust trend of low correlation of the current beat against the reference template together with shortened current RR-interval duration for VB-class. There is one more feature interaction (F15*F18), which is common for 3 of the classification models (Cluster, Fuzzy, CT) that combines the estimation of slower edge/larger area QRS morphologies with shortened current RR-interval duration for VB-class enhancement. We observe that there are features which alone do not contribute robustly to the separation of SVB vs. VB class, however, they are factors in several top-10 interaction effects, i.e: F1 (template type matched by current beat), F4 and F5 (P-wave presence in current beat and reference template), F7 (correlation of previous beat to reference template). Among all features, we can highlight the three best scored ones with 11, 9 and 8 interactions selected in top-10 by all 4 classifiers: F18 (current RR-interval duration), F15 (beat QRS mobility), F6 (beat correlation).

## Discussion

Automatic detection and classification of heartbeats is an important computerized diagnostic tool applied in real-time monitoring applications and for assisting cardiologists in the task of long-term ECG inspection by marking the presence of sustained, transient or casual arrhythmias, as well as for a reliable counting of ventricular extrasystoles in exercise testing. We implement a two-stage heartbeat classification platform with a simple first stage and a precise second stage, particularly advantageous for real-time applications. About 92.8% (1069869/1152964) of all beats in the training dataset are immediately assigned to SVB-class by computationally efficient Stage 1, using a simple correlation threshold criterion for finding a close match with the reference template of the patient’s predominant rhythm. The most resource-consuming task is the measurement process of 20 basic features that is, however, simplified by calculations in the time-domain for the few residual non-matched beats (7.2%, 83095/1152964 beats) which are fed to Stage 2 for a subsequent refined classification in SVB or VB-class. Stage 2, however, takes a delayed decision after the features of the next beat have been acquired.

The most important design considerations for Stage 1 as a PQRST waveform preprocessor are:

- Template matching conditions–an adaptive correlation threshold, optimized to the current noise conditions is preferred as a fast-computation estimator of the PQRST waveform similarity to the reference template. This relatively simple criterion achieves detection at Sp = 95.3%, Se = 93.9% (training) and 91.2%, 94.4% (test-validation), however, at very low PPV = 36.3% (training) and 48.1% (test-validation)–see Table 2. This indicates for a great inconformity between the numbers of FN and FP errors due to the imbalanced number of SVB-class and VB-class beats in the learning dataset, and points to the need for a subsequent heartbeat classification step dedicated to reduction of the FP rate. Stage 1 has the disadvantage to limit the overall sensitivity of Stage 1 + Stage 2 since about 6% of all VB-beats will be erroneously classified as SVB-beats by Stage 1 and will not be analysed by Stage 2.
- Set of heartbeat features–a set of 20 basic features with a physiological meaning (morphological similarity with the predominant reference template, P wave existence, QRS complex slope, area and width properties and relative beat timing) are extracted by Stage 1 following three main concepts: (i) morphological and RR-interval features are calculated for the current PQRST waveform; (ii) morphological and RR-variability features are calculated for the neighboring beats thus giving information about the ongoing patient’s rhythm; (iii) noise robust morphological features are extracted from continuously averaged reference beat templates. The feature space is extended to a 210-sized vector of the basic 20 features to study their second-order interactions. It makes sense to provide them in parallel and let the subsequent Stage 2 classification method do the optimal selection in an automatic way.

Stage 2 is designed as a classification system with high reliability in categorizing SVB and VB beats, embodying independent realizations of four classification methods: Cluster, Fuzzy, LDA and CT models. The optimal setting of each classifier configuration is derived by supervised learning on a training dataset (AHA+SVDB+VDB) and a vector of 210 heartbeat features. The training process iteratively enters new features in the model following the optimization criterion for minimizing the number of errors–both FP and FN, considered at equal weight that is particularly assessed by maximizing the mean value of Se and PPV. Since PPV is sensitive to data distribution, its presence in the optimization criterion guarantees the effective evaluation of the classification performance in our imbalanced learning scenario.

The training process of Cluster, Fuzzy and LDA models shows that their performance improves until a specific number of features are entered in the model, and any further increase of complexity leads to performance saturation (Cluster–Fig 4, LDA–Fig 7) or degradation (Fuzzy–Fig 6). The best performance solutions for the three models are listed below in ascending order of their training performance [Mean(Se,PPV), Se]:

- Cluster analysis with 9 clusters in 30 dimensional feature space (Figs 4 and 5)–[90.9%, 91.4%];
- Fuzzy analysis with 72 features (Fig 6)–[93.1%, 95.7%];
- LDA function with 142 coefficients (Figs 7 and 8)–[94%, 96.1%];

The training process of CT stops at maximal splitting level, where the highest performance of the model is reported. CT performance can be easily configured by setting different complexity of the model (i.e. by pruning the tree to different levels). For the aims of the comparative study according to cluster, fuzzy and LDA models complexity, CT is pruned to 30, 72, 142 decision nodes (Fig 9). The training performance [Mean(Se,PPV); Se] is as follows:

- CT with 30 decision nodes: [95.3, 96.9%]–better than Cluster analysis by [4.4%; 5.5%];
- CT with 72 decision nodes: [96.8, 97.4%]–better than Fuzzy analysis by [3.7%; 1.7%];
- CT with 142 decision nodes: [98.5, 99%]–better than LDA by [4.5%; 2.9%];
- CT with 221 decision nodes (no pruning): [99.4; 99.7%]–better than Cluster analysis by [8.5; 8.3%], Fuzzy analysis by [6.3; 4%], LDA by [5.4; 3.6%]. Besides, when CT model complexity is increased from 30 to 210 nodes, its performance improves by [2.8; 4.3%].

Comparison of the presented combined classifier (Stage 1+Stage 2) to other published heartbeat classification methods based on cluster, fuzzy, LDA and CT models is shown in Table 5, considering the test-validation performance on MIT-BIH database (usually applied for testing), as well as reporting Sp over N+S beats (SVB-class), and Se, PPV only for V-beats (part of VB-class) by excluding the beats annotated as F-beats from the analysis. The performance on F-beats as part of VB-class is usually not reported in the literature, suggesting their hybrid nature, which drops Se by 1% to 5% (as shown in Table 3, V-beats vs. V+F beats). Considering Sp values of other published studies (80% to 99.8%), our combined classifier has comparable or outperforming Sp (99.4% to 99.9%), which is an effect from the design optimization criterion for minimizing the number of both FP and FN errors during training. This is also evident by the high PPV of this study (92.4% to 99.2%) compared to reported values (71.1% to 99.2%), where available. The negative effect is, however, the decreased Se due to the higher number of FNs (comparable to the number of FPs which actually are small percentage from the largest SVB-class). We rate Stage 2 classifiers in ascending order of their Se–cluster (92.4%), LDA (94.2%), fuzzy (94.4%), CT (96.7%), considering that all fall within the wide range of reported Se (77.7% to 100%) but we highlight CT, being in the top Se scores.

**Table 5:** Performance of the combined beat classifier vs. published studies, using the same linear-programming based classification methods as those implemented in Stage 2. The values of Sp, Se, PPV are shown as reported by the authors, replaced by ‘-‘ mark when not published.

Methods	Feature set	Nb features	Sp (%)	Se (%)	PPV (%)	Test database
**Cluster analysis**						
**Stage 1 + Stage 2**	Morphology, RR intervals, template correlation	30	**99.5**	**92.4**	**93.6**	MIT-BIH
Christov et al. (2005) [9]	Morphology	26	97.7	97.3	-	MIT-BIH
Christov et al. (2006) [5]	Morphology	26	99.1	96.3	89.9	MIT-BIH
Christov et al. (2006) [5]	Matching Pursuits expanding coefficients	110	99.1	94.8	89.2	MIT-BIH
Rodriquez-Sotelo et al. (2012) [10]	Morphology, RR intervals, DWT coefficients, Hermite coefficients	100	95.8	96.1	-	MIT-BIH
Kutlu and Kuntalp (2011) [11]	Morphology, DFT coefficients, WPD coefficients	64	99.8	97.0	99.2	MIT-BIH
Kutlu and Kuntalp (2012) [26]	WPD coefficients	28	96.8	87.6	88.1	MIT-BIH
**Fuzzy analysis**						
**Stage 1 + Stage 2**	Morphology, RR intervals, template correlation	72	**99.4**	**94.4**	**92.4**	MIT-BIH
Wieben et al. (1999) [21]	Filter bank features	9	-	81.3	80.6	MIT-BIH
Behadada and Chikh (2013) [12]	Morphology, RR intervals	10	80.0	100	71.1	MIT-BIH
Krasteva and Jekova (2007) [4]	Morphology, RR intervals, template correlation, filter banks features	5	97.9	98.4	-	MIT-BIH
**Discriminant analysis**						
**Stage 1 + Stage 2**	Morphology, RR intervals, template correlation	142	**99.5**	**94.2**	**93.0**	MIT-BIH
de Chazal and Reilly (2006) [3]	Morphology, RR intervals, global classifier	48	98.8	77.7	81.9	MIT-BIH
de Chazal and Reilly (2006) [3]	Morphology, RR intervals, local classifier	48	99.7	94.3	96.2	MIT-BIH
Jekova et al. (2004) [13]	Morphology	18	97.3	93.3	-	MIT-BIH
García et al. (2011) [14]	Morphology, RR intervals	12	97.2	97.7	-	MIT-BIH SVDB
Llamedo and Martinez (2011) [15]	DWT, Morphology, RR intervals	8	95.0	81.0	87.0	MIT-BIH INCART
**Classification tree**						
**Stage 1 + Stage 2 (221 nodes)**	Morphology, RR intervals, template correlation	203	**99.9**	**96.7**	**99.2**	MIT-BIH
Wieben et al. (1999) [21]	Filter bank features	14	-	85.3	85.2	MIT-BIH
Lin and Yang (2007) [16]	Morphology, RR intervals	29	93.0	100	-	MIT-BIH
Mert et al. (2012) [17]	Morphology, RR intervals, Single decision tree	6	98.4	96.1	95.6	MIT-BIH
Mert et al. (2012) [17]	Morphology, RR intervals, Majority voting of 65 bootstrap decision trees	6	99.5	96.7	98.3	MIT-BIH

The comparison with other studies that apply the same classification methods gives us the opportunity to weight the advantages/disadvantages of the selected feature set. Although the notable disadvantage of larger feature set for LDA, Fuzzy and CT models than the compared studies, it should be underlined that they are derivatives from 20 basic time-domain features so that the feature extraction complexity is simple. Having a look at the feature space in the works, reporting higher Sp, Se, PPV than our respective methods (Table 5), we can highlight [3] with discriminant analysis over 48 morphology and RR-interval features and [11] with Knn classifier over 64 time, frequency and time-frequency domain features. By means of feature space similar to our study, the achieved better performance in [3] is valid when a local but not a global learning strategy is applied. The insight over the heartbeats in 3 domains (3D) by combining morphological features, energy spectral density by Fourier transform and higher order statistics of wavelet decomposition coefficients in [11] seems to contribute for better V-beats clusterization than a reduced set of only 28 time-frequency domain features reported by the same authors in [26] and 30 time-domain features when using the Cluster analysis in this study. The CT model with 203 time-domain features is, however, able to achieve competitive performance to the Knn classifier with 3D feature set [11].

Although the use of standardized training and test databases allows comparison between different studies (Table 5), a possible limitation is that they might not match the clinical setting. It is expected lower occurrence of V-beats and this might lead to decrease in Sp and PPV, while keeping Se.

## Conclusions

This study shows successful strategies for building a computationally-efficient and reliable classifier of SVB and VB beats, with special considerations for real-time application:

1. Simple first stage classifier using only one feature–an adaptive correlation threshold, optimized to the current noise conditions classifies about 93% of the beats in SVB-class whose PQRST waveform is highly correlated with the reference template of the patient’s predominant rhythm;
2. Simplified feature extraction process, which is run on a reduced set of not matched beats (only about 7% of all detected beats) and is required to calculate for them only 20 time-domain features of the morphological and RR-variability behavior of the single beat and the averaged noise robust reference template;
3. Second-stage classifier with embedded CT-model shows the top-ranked values of Sp = 99.9%, Se = 96.7%, PPV = 99.2%, compared to other linear classifiers (fuzzy, cluster, discriminant) and other published studies on MIT-BIH database. Indeed, we recommend the decision tree as the ultimate prediction model that best fits the requirements in clinical practice–high performance, flexibility and easy adjustment of the error rates for different beat classes (e.g. less false alarms).

While the final goal of a beat classification algorithm is to distinguish between (N,S,V,F) beats, in this study we focus on the reduced binary class model (SVB and VB) as this is a clinically relevant pre-step of the (N,S,V,F) classification problem that distinguishes between beats of normal morphology (narrow supraventricular beats) and abnormal morphology (dangerous wide ventricular beats).
